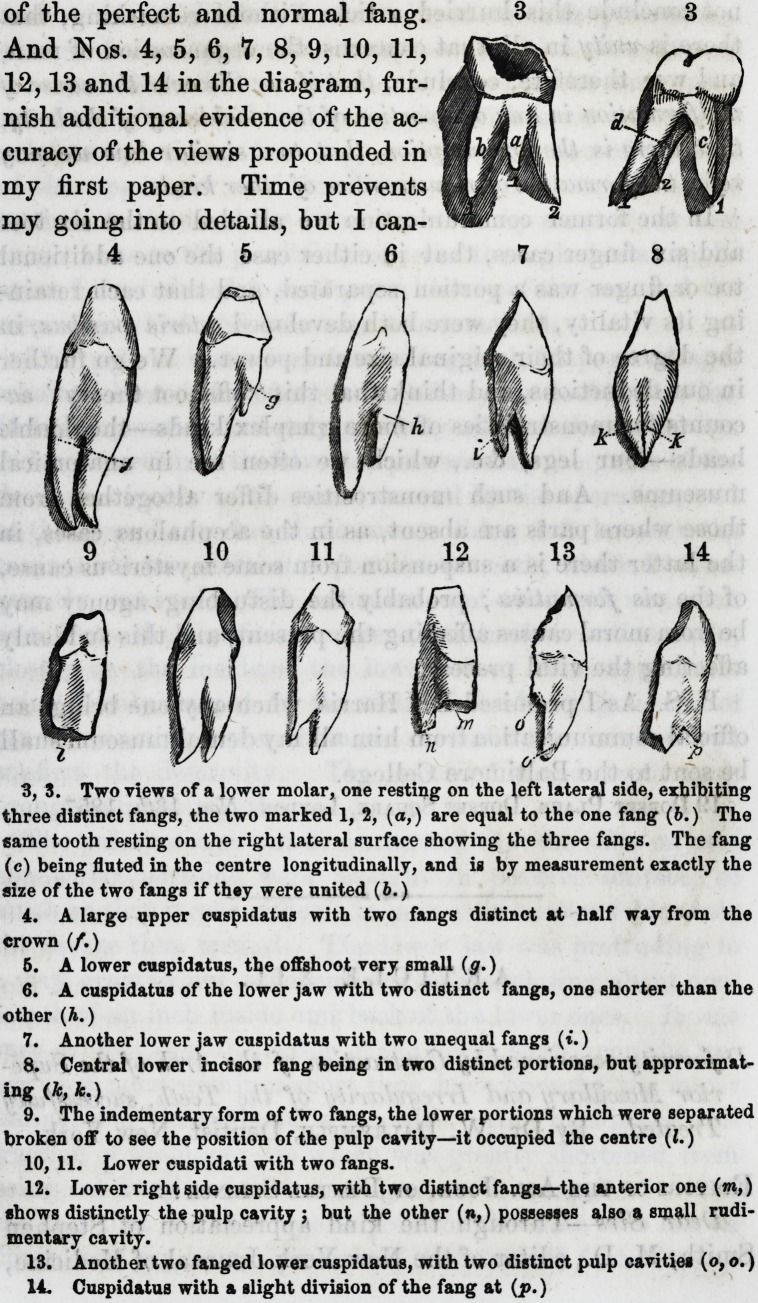# Second Paper on Supernumerary Teeth, with a Few Remarks on Monstrosities

**Published:** 1858-01

**Authors:** J. L. Levison


### ARTICLE XI.

*Second Paper on Supernumerary Teeth, with a few Remarks on Monstrosities.* By J. L. LEVISON, D. D. S., &c., &c.

SIRS:—It may be regarded as an important truth that every actual discovery in the physical sciences, in moral science, and in physiology, is rendered obvious by every subsequent investigation. Nor does it alter the conclusiveness of this statement, whether the *new* law propounded, has been the result of many experiments, or the immediate induction is founded on some few observations, or by what is called "an accident," if it is an exposition of some principle it will have the advantage of being obvious so soon as it is put to the test. And, further, that the test of the accuracy of the induction is confirmed by every subsequent investigation.

With these premises, I would submit to you a series of *diagrams* with explanations, as they furnish collateral evidence of the accuracy of my former observations on the *modus operandi* by which supernumerary teeth are formed. And I may venture to speculate, that simple as this discovery is, that it furnishes some *data* to explain the *law* of monstrosities in general.

If we may judge of the importance of *little* things compared to great ones, we may digress a few moments to mention that it is well known that Sir Isaac Newton had been pondering for years to ascertain the law which held the planets in their course, and sustained them in their orbits, but that he made his discovery in an instant by seeing an apple fall to the ground, and by its means he unfolded the law of gravitation! And all his subsequent investigations tended to elucidate the accuracy of his first inference.

Now every insight into the *arcana* of nature, display the marvellous wisdom of the creator, and it is difficult to draw the line of demarcation, as applied to any one of the said laws—small and great being but relative terms, and hence the skill and perfection of the Deity is shown equally in minute as in the vast objects of his creative power!

After these commentaries, I may allege that a *small* subject, namely—the law by which supernumerary teeth are formed "that they were offshoots, and that they continued to grow, possessing the *vis formatio*, and so forth." Now in proof of the accuracy of these views we submit the annexed *diagrams* of certain *lusus naturæ*, so called from their having more than the usual number of fangs. And we now call the attention of your scientific readers to the special fact, that each of the specimens figured confirm the theory of offshoots from the pulps, as seen in No. 1, and the separation of in diagram No. 2, and lastly the two views of diagram 3, 3, in which the additional fang is proved to be but a part of his neighbor, inasmuch that the two smaller ones are just in volume the size and weight

1 2

*Explanation of the Diagrams.*

**1. Lower jaw molar necrosed from the right lateral and anterior surface, showing the broad anterior fang on the one side (A,) which is just as broad as the two on the posterior side (B.)**

**2. Bicuspid of the upper jaw with three fangs. The two on the anterior surface (*a*,) equal in volume and breadth to the single posterior fang (*b*.)**

3 3

of the perfect and normal fang. And Nos. 4, 5, 6, 7, 8, 9, 10, 11, 12, 13 and 14 in the diagram, furnish additional evidence of the accuracy of the views propounded in my first paper. Time prevents my going into details, but I can-

4 5 6 7 8

9 10 11 12 13 14

**3, 3. Two views of a lower molar, one resting on the left lateral side, exhibiting three distinct fangs, the two marked 1, 2, (*a*,) are equal to the one fang (*b*.) The same tooth resting on the right lateral surface showing the three fangs. The fang (*c*) being fluted in the centre longitudinally, and is by measurement exactly the size of the two fangs if they were united (*b*.)**

**4. A large upper cuspidatus with two fangs distinct at half way from the crown (*f*.)**

**5. A lower cuspidatus, the offshoot very small (*g*.)**

**6. A cuspidatus of the lower jaw with two distinct fangs, one shorter than the other (*h*.)**

**7. Another lower jaw cuspidatus with two unequal fangs (*i*.)**

**8. Central lower incisor fang being in two distinct portions, but approximating (*k*, *k*.)**

**9. The indementary form of two fangs, the lower portions which were separated broken off to see the position of the pulp cavity—it occupied the centre (*l*.)**

**10, 11. Lower cuspidati with two fangs.**

**12. Lower right side cuspidatus, with two distinct fangs—the anterior one (*m*,) shows distinctly the pulp cavity; but the other (*n*,) possesses also a small rudimentary cavity.**

**13. Another two fanged lower cuspidatus, with two distinct pulp cavities (*o*, *o*.)**

**14. Cuspidatus with a slight division of the fang at (*p*.)**

not conclude this hurried article without remarking, that there is *unity* in all that concerns the organization of man, and we, therefore, conclude, *that if we discover the cause of malformation in any one portion of the machinery of the body, that there is the presumption, that to a similar law we may refer the formation of monstrosities of other kinds.*

In the former communication we alluded to the six toes and six finger cases, that in either case the one additional toe or finger was a portion separated, and that each retaining its vitality, they were both developed *ceteris paribus*, in the degree of their original size and power. We go further in our deductions, and think that this "offshoot theory" accounts for monstrosities of more complex kinds—the double heads—four legs, &c., which we often see in anatomical museums. And such monstrosities differ altogether from those where parts are absent, as in the acephalous cases, in the latter there is a suspension from some mysterious cause, of the *vis formativa;* probably the disturbing agency may be from moral causes affecting the present, and this suddenly affecting the vital process.

P. S. As I promised Dr. Harris, when any one brings an official communication from him all my dental museum shall be sent to the Baltimore College.

19 DORSET PLACE, DORSET SQUARE, LONDON, *Nov.* 13*th*, 1857.

## Figures and Tables

**Figure f1:**
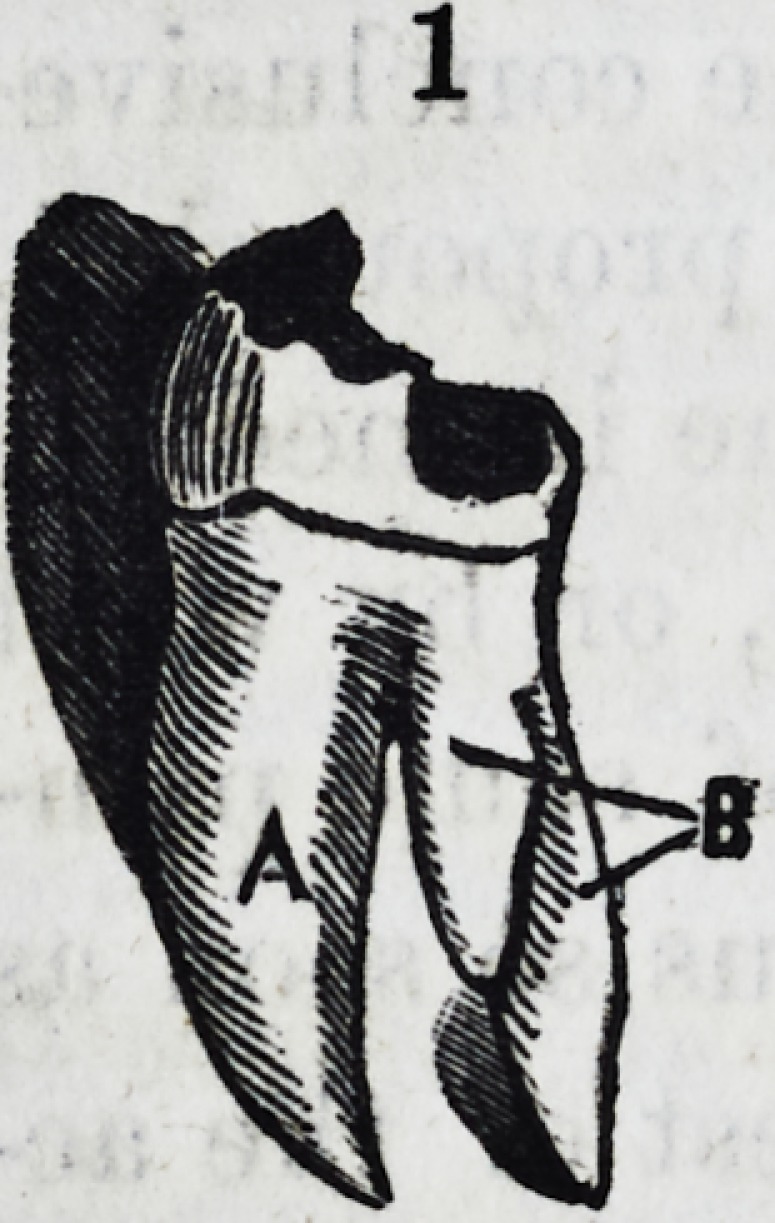


**Figure f2:**
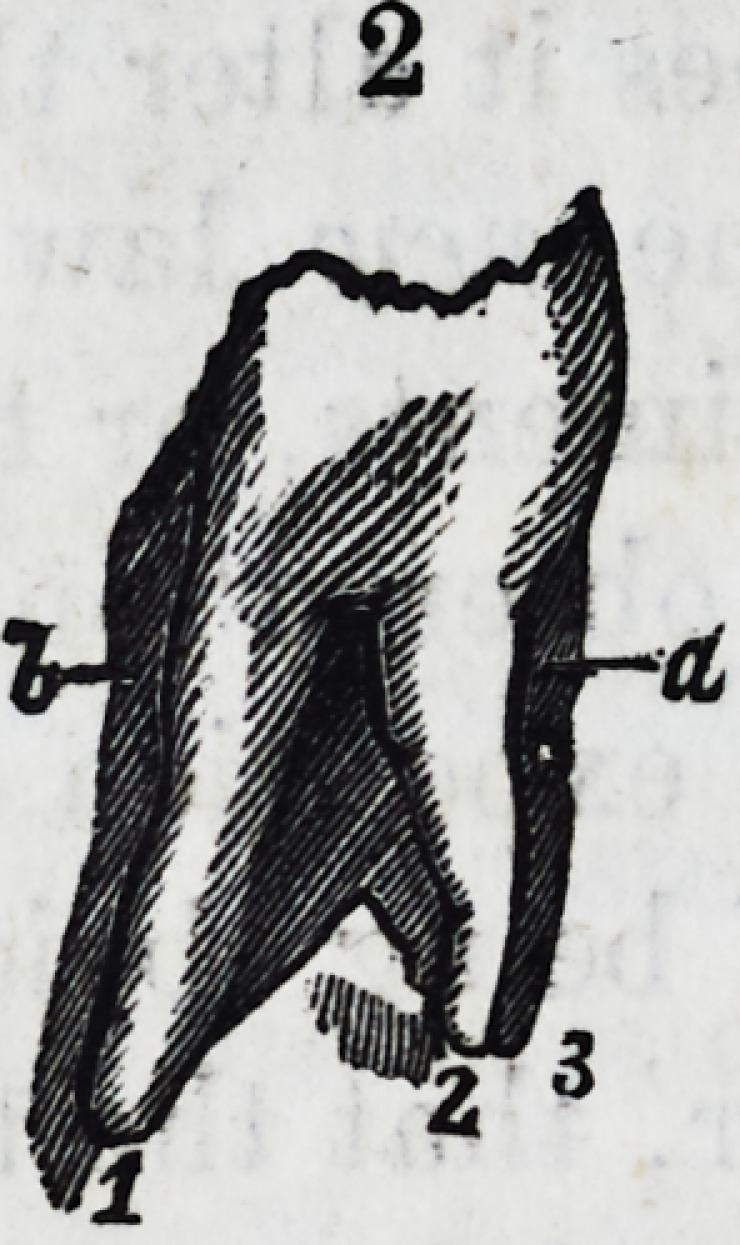


**Figure f3:**