# Typographical Error

**Published:** 1858-04

**Authors:** J. L. Levison


*Typographical Error.*—We insert, with pleasure, the following correction of a typographical error which occurred in our last number.

Eds.

19 Dorset Place, Dorset Square, London, N. W.
February 19th, 1858.

Messrs. Editors:—May I ask you to correct an error, which interferes with the sense of the concluding paragraph of my last article in the Journal for January, page 56. It stands thus: "probably the disturbing agency may be from moral causes affecting the present." The latter word should have been "parent;" for only through the mother, can the fœtus in utero, have the vital process affected.

I am, my dear sirs, in haste,
Yours, very truly,

J. L. Levison.

